# What neurological and psychiatric effects does COVID-19 have on its survivors?

**DOI:** 10.1186/s12916-021-01986-7

**Published:** 2021-04-29

**Authors:** Alexios-Fotios A. Mentis

**Affiliations:** 1 University Research Institute of Maternal and Child Health & Precision Medicine, Athens, Greece; 2 UNESCO Chair on Adolescent Health Care, National and Kapodistrian University of Athens, “Aghia Sophia” Children’s Hospital, Athens, Greece

## Background

Since the COVID-19 pandemic began, there has been a steadily increasing interest on the COVID-19 infection’s neurological and psychiatric sequelae, such as dementia and mood/anxiety disorders [1]. However, the majority of studies reporting on COVID-19−related neurological complications are generally confined to small series of patients and to small numbers of hospitals and are limited to locations and specialization; thus, the neuropsychiatric consequences of COVID-19 are not evaluated across a broader spectrum of population and independent geographic settings [2–4]. Hence, larger, more robust, and long-term data are essential to define and critically evaluate the effects of the COVID-19 pandemic on neurological and psychiatric disorders. This information is of paramount importance for both the planning of health services and the identification of key research priorities.

## Main text

In this context, Taquet and colleagues recently assessed the prevalence of neurological and mental health diagnoses in 6-month survivors of COVID-19 infection, as well as the associated hazard ratios in comparison to other health conditions. To this end, Taquet and colleagues used a well-defined and quantified large-scale, stable, and prolonged data from the TriNetX Analytics Network, Cambridge, USA (covering over 81 million patients from the USA and Europe, the Middle East, and Africa countries). According to the study, 33.6% of COVID-19 patients reported neurological and psychiatric complications. Nonetheless, psychiatric diagnosis was less in association with COVID-19 than neurological diagnosis.

Neurologists and mental health specialists face many critical research questions during the COVID-19 pandemic; for instance, (a) what is the occurrence rate of neurological outcomes in survivors of COVID-19 infection, and the associated hazard ratios in comparison to other health conditions, i.e., influenza or other respiratory infection; and (b) what population groups are at most danger? Early clinical neurological manifestations of COVID-19 have not been widely documented in the literature, at least in part because most patients with COVID-19 were treated by medical doctors who are specialized in other fields, such as internal medicine and intensive care unit specialists [3]. More extensive and integrated epidemiological characterization is vital if the processes underlying those presentations are to be understood without which suitable treatments cannot be rationally selected, evaluated, and used.

Taquet and colleagues measured the occurrence of (a) intracranial hemorrhage, (b) ischemic stroke, (c) Parkinson disease, (d) Guillain-Barré syndrome, (e) nerve disorders, (f) myoneural disease, (g) dementia, (h) anxiety/mood, (i) encephalitis, (j) psychotic disorders, (k) insomnia, and (l) substance misuse, in patients with documented COVID-19, and the associated hazard ratios in comparison to other health conditions, i.e., influenza or other respiratory infection, by the use of retrospective cohort studies and time-to-event analysis (*a method for analyzing the length of time until the occurrence of an event of interest*, i.e., Cox model). In comparison to matching cohorts with influenza and other respiratory infection, the occurrences of neurological outcomes are significantly higher in patients with COVID-19 infection, except those with Parkinson disease and Guillain-Barré syndrome. The study also reported the significant ties between COVID-19 infection and dementia, compared to influenza or other respiratory infections.

The authors also explored how the neurological and mental health consequences of COVID-19 varied, depending on the severity of diseases mainly in those who required hospitalization or had encephalopathy during the COVID-19 infection, and how the hazard ratios changed over a period of 24 weeks. The incidences of neurological diagnosis and the hazard ratios were increased in patients who needed hospitalization and significantly higher in those who developed encephalopathy. Furthermore, when compared to neurological disorders, the hazard ratios for psychiatric disorders, such as anxiety and mood disorders, showed a less strong association with encephalopathy and hospitalization. This observation suggests that mental health deterioration due to social distancing and pandemic-related anxiety may have partly contributed to psychiatric illness; thus, it is unlikely they represent a direct manifestation of the COVID-19 illness. Moreover, the underlying mechanisms for such associations include direct effects of viral tropism on the central nervous system, angiotensin-converting enzyme-2 receptor-related neuronal damage, cytokine overproduction and toxicity-related neuronal injury, and immune response-related neuronal injury [1, 5].

Taquet and colleagues also emphasize the importance of interdisciplinary work in the field of clinical neuroscience, especially during the COVID-19 period. Survivors from COVID-19 infection tend to be at higher risk of psychiatric consequences, and the risk of COVID-19 for patients who have acute neurological or mental syndromes should be warned by medical practitioners. As a result, clinicians should be cautious about how COVID-19 infection is linked to neurological and psychiatric disorders, given that the altered mental state is the second most common presentation in patients with COVID-19 infection after respiratory illness, in particular in younger patients [5]. This study provides important and timely information that physicians, investigators, and funders may need to guide immediate action in neuroscience research for COVID-19 patients and guide health policy for COVID-19 management. Albeit a broad, global, diverse, and cross-speciality collaborative network, there are some drawbacks in the study which are, however, inherent to an electronic health records report; these include unknown record completeness, lack of diagnostic validation, and scarce socioeconomic and lifestyle information. Moreover, delirium and other altered mental conditions are well-established conditions in the acute disease setting. In contrast, the cases discussed by Taquet and colleagues may have presented distinct characteristics. Therefore, outcomes for this population should not be considered generalizable to all delirium patients [2]. Last, such a study design may only depict associations; attempts to classify pathways and measure causality will require more detailed study designs [2].

## Conclusion

In conclusion, the study by Taquet et al. indicates severe neurological and psychiatric illness following infection with COVID-19. The knowledge reaped from this study can assist in health services planning and in identifying future research goals. Globally, the cohort approach reported offers useful, timely, and critically required information for other researchers and funding providers to guide the future steps in the COVID-19 research related to clinical neurosciences and mental health strategy.

## Data Availability

Not applicable.
